# Supplementary material for: Etiology of acute febrile illnesses in Southern China: Findings from a two-year sentinel surveillance project, 2017–2019
Source: PLoS One. 2022 Jun 28;17(6):e0270586. doi: 10.1371/journal.pone.0270586 (PMC9239456; doi:10.1371/journal.pone.0270586)
Supplement: S1 File — (DOCX) [file pone.0270586.s001.docx]

**S1 Supplement**

Table A. Co-infections and cycle threshold values (Ct)* detected from TaqMan Array Card (TAC) diagnostic test for patients enrolled in the acute febrile illness surveillance project by location in China, June 2017 – July 2019.

| **Location** | **Pathogen 1 (Ct value)** | **Pathogen 2 (Ct value)** |
| --- | --- | --- |
| **Jiangmen City** | C. burnetti (31.7) | Rickettsia (32.4) |
|  | C. burnetti (30.5) | Hepatitis E (34.9) |
| **Mengla County** | O. tsutsugamushi (32.1) | Dengue (34.8) |
|  | O. tsutsugamushi (32.1) | Brucella (34.0) |
|  | O. tsutsugamushi (26.4) | Brucella (34.4) |
|  | O. tsutsugamushi (24.0) | Brucella (34.0) |
|  | O. tsutsugamushi (25.8) | Dengue (34.2) |
|  | Brucella (34.5) | Rickettsia (34.8) |

*Pathogen order based on Ct values for each TAC target (target with lowest Ct value listed as pathogen #1).

**Table B**. TaqMan Array Card (TAC) diagnostic test results* for patients enrolled in the acute febrile illness surveillance project by sentinel hospital, China, June 2017 – July 2019.

|  |  | **Enping County, Jiangmen City** | **Shahu Township, Jiangmen City** | **Xinhui District, Jiangmen City** | **Siqian Township, Jiangmen City** | **Mengla County** |
| --- | --- | --- | --- | --- | --- | --- |
|  | **Total** |  |  |  |  |  |
|  | n = 796 | n = 189 | n = 54 | n = 120 | n = 11 | n = 422 |
| **Bacteria** |  |  |  |  |  |  |
| *Brucella* spp. | 14 (2%) | 1 (0.5%) | 0 (0%) | 0 (0%) | 0 (0%) | 13 (3%) |
| *Coxiella burnetiid* | 42 (5%) | 10 (5%) | 1 (0%) | 29 (24%) | 1 (9%) | 1 (0%) |
| *Leptospira* spp. | 5 (1%) | 2 (1%) | 0 (0%) | 0 (0%) | 0 (0%) | 3 (1%) |
| *Orientia tsutsugamushi* | 60 (8%) | 9 (5%) | 2 (4%) | 10 (8%) | 2 (18%) | 37 (9%) |
| *Rickettsia* spp. | 9 (1%) | 1 (1%) | 0 (0%) | 0 (0%) | 0 (0%) | 8 (2%) |
| *Streptococcus suis* | 1 (0%) | 0 (0%) | 0 (0%) | 1 (1%) | 0 (0%) | 0 (0%) |
| *Salmonella* Typhi | 2 (0%) | 0 (0%) | 0 (0%) | 1 (1%) | 0 (0%) | 1 (0%) |
| **Viruses** |  |  |  |  |  |  |
| Dengue virus | 205 (26%) | 13 (7%) | 1 (0%) | 2 (2%) | 0 (0%) | 189 (45%) |
| Hepatitis E | 1 (0%) | 0 (0%) | 0 (0%) | 1 (1%) | 0 (0%) | 0 (0%) |
| **Protozoa** |  |  |  |  |  |  |
| *Plasmodium* spp. | 10 (1%) | 8 (4%) | 1 (0%) | 1 (1%) | 0 (0%) | 0 (0%) |
| **No pathogen detected** | 455 (57%) | 146 (77%) | 49 (91%) | 76 (63%) | 8 (73%) | 176 (42%) |

*The number of TAC results is greater than the number of enrolled patients due to the eight patients who had co-infections with two of the pathogens on TAC.

**Table C.** Association between patient characteristics/epidemiologic factors and detection of at least one pathogen on the TaqMan Array Card (TAC)* diagnostic testing platform, China, June 2017 – July 2019.

| **Characteristic/Epidemiologic risk** | **Any TAC+ Result** | **Crude OR (95% CI)** | **ORa (95% CI)** |
| --- | --- | --- | --- |
|  | **n (%)** |  |  |
| **Location** |  |  |  |
| Mengla County | 246 (72.1%) | 4.1 (3.0-5.6) | 5.5 (3.8-8.0) |
| Jiangmen City | 95 (27.9%) | Ref | Ref |
| **Nationality** |  |  |  |
| Other | 12 (3.5%) | 1.1 (0.5-2.5) | 1.1 (0.4-3.2) |
| Chinese | 329 (96.5%) | Ref | Ref |
| **Sex** |  |  |  |
| Male | 178 (52.2%) | 0.8 (0.6-1.0) | 0.9 (0.6-1.2) |
| Female | 163 (47.8%) | Ref | Ref |
| **Age group** |  |  |  |
| >=18 years | 297 (87.1%) | 2.9 (2.0-4.3) | 1.6 (0.7-3.3) |
| 2-17 years | 44 (12.9%) | Ref | Ref |
| **Occupation** |  |  |  |
| Farmer, manufacturing, fisherman | 134 (39.3%) | 2.6 (1.5-4.8) | 0.9 (0.5-1.9) |
| Transportation (e.g., taxi/bus driver) | 6 (1.7%) | 4.4 (1.0-22.7) | 4.4 (0.9-23.6) |
| Office worker | 20 (5.9%) | 1.8 (0.8-4.1) | 1.3 (0.5-3.2) |
| Student | 39 (11.4%) | 0.6 (0.3-1.2) | 0.4 (0.1-1.0) |
| Other | 123 (36.1%) | 2.1 (1.2-3.9) | 1.3 (0.7-2.5) |
| Homemaker | 19 (5.6%) | Ref | Ref |
| **Education** |  |  |  |
| Less than primary school | 7 (2.1%) | 2.6 (0.9-7.4) | 0.6 (0.1-2.2) |
| Primary school | 91 (26.7%) | 3.7 (2.3-5.9) | 1.1 (0.4-3.1) |
| High school | 152 (44.6%) | 2.8 (1.8-4.3) | 1.1 (0.4-2.9) |
| College | 52 (15.2%) | 3.1 (1.9-5.3) | 1.0 (0.3-2.9) |
| Currently in school | 39 (11.4%) | Ref | Ref |
| **Travel prior to onset** |  |  |  |
| No | 313 (91.8%) | 0.9 (0.6-1.6) | 0.6 (0.3-1.1) |
| Yes | 28 (8.2%) | Ref | Ref |

Includes 27 unique targets: Bartonella, Brucella, Burkholderia pseudomallei, Coxiella burnetii, Chikungunya, Bundibuygo & Sudan, Dengue, Leptospira, Lassa, Nipah, Orientia tsutsugamushi, Plasmodium, Rickettsia, Salmonella, Salmonella Typhi, Salmonella Paratyphi A, Streptococcus pneumoniae, Streptocococcus suis, T. brucella, Yersina pestis, Zika virus, CCHF, Ebola, Hepatitis E, Leptospira, Mayaro, Marburg & O'noyong-nyong, P. falciparum/vivax, Rift Valley Fever, *Leishmania*, Yellow Fever.

Table D. Clinical characteristics of the six main pathogens detected by the TaqMan Array Card diagnostic test* among patients presenting with acute febrile illnesses, Jiangmen City and Mengla County, China, June 2017 – July 2019.

| **Clinical Factors** | **Dengue** | ***O.*** ***tsutsugamushi*** | ***C. burnetii*** | ***Plasmodium* spp.** | ***Brucella*** **spp**. | ***Rickettsia*** **spp**. | **Total** |
| --- | --- | --- | --- | --- | --- | --- | --- |
|  | n = 205 | n = 60 | n = 42 | n = 10 | n =14 | n = 9 | n = 333 |
| Received Blood | 2 (1%) | 1 (2%) | 0 (0%) | 0 (0%) | 0 (0%) | 0 (0%) | 3 |
| Rash | 70 (34%) | 8 (13%) | 1 (2%) | 1 (10%) | 0 (0%) | 1 (11%) | 81 |
| Redeyes | 8 (4%) | 3 (5%) | 2 (5%) | 0 (0%) | 0 (0%) | 2 (22%) | 15 |
| Joint pain | 65 (32%) | 14 (23%) | 4 (10%) | 1 (10%) | 4 (29%) | 1 (11%) | 89 |
| Headache | 141 (69%) | 41 (69%) | 24 (57%) | 6 (60%) | 11 (79%) | 6 (67%) | 229 |
| Chills | 68 (33%) | 41 (68%) | 26 (62%) | 5 (50%) | 9 (64%) | 6 (67%) | 155 |
| Muscle Pain | 107 (52%) | 31 (52%) | 14 (33%) | 2 (20%) | 6 (43%) | 5 (56%) | 165 |
| Vomiting | 12 (6%) | 6 (10%) | 4 (10%) | 0 (0%) | 4 (29%) | 1 (11%) | 27 |
| Bloody Sputum | 1 (0%) | 1 (2%) | 0 (0%) | 0 (0%) | 0 (0%) | 0 (0%) | 2 |
| Bone Pain | 19 (9%) | 6 (10%) | 2 (5%) | 1 (10%) | 2 (15%) | 0 (0%) | 30 |
| Nose/Gum Bleeding | 7 (3%) | 0 (0%) | 3 (7%) | 0 (0%) | 0 (0%) | 0 (0%) | 10 |
| Swollen Joints | 2 (1%) | 2 (3%) | 1 (2%) | 0 (0%) | 1 (7%) | 0 (0%) | 6 |

*The number of TAC results is greater than the total patients with a positive result for one of the six main pathogens due to the seven patients who had co-infections with two of the six main pathogens on TAC.

**Table E.** Comparison of Tac Man Array Card (TAC) results and rapid diagnostic test (RDT) for dengue for AFI patients enrolled at sentinel hospitals in Mengla County, Yunnan and Jiangmen City, Guangdong, China, 2017 – 2019.

| **Test Platform/Hospital Location** | Dengue NS1+ | Dengue NS1- | Cohen's Kappa* |
| --- | --- | --- | --- |
| All hospitals combined (n = 1,866) |  |  |  |
| TAC+ | 174 (23%) | 26 (3%) | 0.84 (95% CL: 0.8 - 0.9) |
| TAC - | 21 (3%) | 550 (71%) |  |
| Jiangmen City (n = 417) |  |  |  |
| TAC+ | 0 (0%) | 18 (5%) | 0.009 (95% CL: -0.5 - 0.4) |
| TAC- | 2 (1%) | 372 (94%) |  |
| Mengla County (n = 449) |  |  |  |
| TAC+ | 177 (44%) | 10 (3%) | 0.85 (95% CL: 0.8 - 0.9) |
| TAC- | 19 (5%) | 196 (49%) |  |

*Cohen’s Kappa interpretation: If kappa is less than 0, "No agreement", if 0-0.2, "Slight agreement", if 0.2-0.4, "Fair agreement", if 0.4-0.6, "Moderate agreement", if 0.6-0.8, "Substantial agreement", if 0.8-1.0, "Almost perfect agreement".
